# Overcoming Infertility Challenges: A Case Report on the Management of Ovarian Endometriomas and Successful Pregnancy With Intracytoplasmic Sperm Injection and Platelet-Rich Plasma Perfusion

**DOI:** 10.7759/cureus.56468

**Published:** 2024-03-19

**Authors:** Priyal Tilak, Pranita A Bawaskar, Ankit Badge, Nancy Nair, Avanti Kalbande, Pranjali P Muley

**Affiliations:** 1 Clinical Embryology, School of Allied Health Sciences, Datta Meghe Institute of Higher Education & Research, Nagpur, IND; 2 Microbiology, Datta Meghe Medical College, Datta Meghe Institute of Higher Education & Research, Nagpur, IND; 3 Obstetrics and Gynaecology, Datta Meghe Medical College, Datta Meghe Institute of Higher Education & Research, Nagpur, IND; 4 Physiology, Datta Meghe Medical College, Datta Meghe Institute of Higher Education & Research, Nagpur, IND

**Keywords:** dyspareunia, dysmenorrhea, primary infertility, transvaginal ultrasound, intracytoplasmic sperm injection, endometriosis

## Abstract

Endometriosis and infertility are clinically associated. The therapeutic approaches for endometriosis, whether medical or surgical, yield distinct outcomes for a woman’s potential for achieving conception, whether through natural means or with the aid of assisted reproductive technology (ART). In this case report, a 29-year-old female and her 32-year-old partner, married for the last five years, sought assistance at our fertility clinic after having one failed in vitro fertilization (IVF) cycle. The patient had a history of dysmenorrhea and deep dyspareunia, suggesting the presence of an ovarian cyst. Transabdominal ultrasound and laparoscopy confirmed the existence of ovaries with adhesions and a chocolate cyst measuring 8 cm × 6 cm in dimensions. Cystectomy of ovarian endometriomas enhances the rate of spontaneous conception and reduces pain. Moreover, it has the potential to enhance the outcome of IVF. The successful outcome achieved through ART, specifically the intracytoplasmic sperm injection cycle, underscores the importance of technological advancements in overcoming infertile barriers. This case report exemplifies the personalized and innovative approaches available to couples undergoing fertility treatment.

## Introduction

Couples seeking infertility treatment are becoming more prevalent worldwide. Infertility is well defined as the inability to achieve a clinical pregnancy following a span of 12 months of consistent and unprotected sexual intercourse. Between 8% and 12% of couples of reproductive age globally are estimated to be affected by it [1]. It has been observed that males alone account for 20-30% of cases of infertility, but they are responsible for 50% of all cases in general [2]. Endometriosis impacts around 10% of women in their reproductive years and 10-25% of individuals undergoing assisted reproductive technology (ART) [3]. It is associated with dysmenorrhea, persistent pelvic pain, and an inability to conceive. Endometriotic ovarian cysts are among the most prevalent forms of the disease, which may be present in up to 20-40% of women with endometriosis undergoing in vitro fertilization (IVF) [4].

Endometriosis typically presents as adhesions, superficial and deep pelvic implants, and ovarian cysts. Laparoscopic investigation is usually necessary for the detection of peritoneal implants and adhesions [5]. IVF offers the highest success rates among ART procedures and is frequently employed in the management of infertility in women with endometriosis [6,7]. The importance of the investigation into this association is increased because the assessment of patients undergoing IVF permits the study of key ovarian indicators of reproductive outcomes, including peak estradiol (E2) levels, oocyte retrieval numbers, fertilization, implantation, and pregnancy rates [8].

It is debatable whether or not laparoscopic removal of ovarian endometriomas improves the likelihood of conception in women chosen for IVF and intracytoplasmic sperm injection (ICSI) cycles. Endometrioma may directly contribute to the distortion of the tubo-ovarian anatomy; however, increased pro-inflammatory cytokine release and oxidative stress may also negatively impact ovarian function, leading to poor folliculogenesis, lower-quality oocytes with decreased fertilization potential, and, ultimately, lower-quality embryos with low implantation potential [9]. Research conducted on females with unilateral disease and comparing the ovarian responses in the affected and contralateral intact gonads suggests that removing endometriomas substantially reduces the quantity of ovarian reserve [10]. Endometrial receptivity and thickness play a vital role in successfully achieving a pregnancy. In women struggling with recurrent implantation failure (RIF) and a thin endometrial lining, intrauterine autologous platelet-rich plasma (PRP) perfusion has been employed [11].

## Case presentation

Couple history

This case report presents the experience of a couple who faced primary infertility with one failed IVF in a previous IVF center and had undergone fertility treatment at our fertility center in Wardha, India, in 2022. The female patient was 29 years old, and her husband was 32 years old. They had been married for five years and had been attempting to conceive for the last three years; the couple underwent a fertility evaluation. She had complained of chronic pelvic pain and dysmenorrhea for the past two years. The pain was progressive, exacerbated during menstruation, and associated with dyspareunia; the patient reported no surgical treatment history. The male partner had a habit of alcohol consumption and smoking occasionally for the past five years. They sought medical assistance due to their inability to conceive despite regular, unprotected intercourse. They did not have a genetic abnormality in themselves or their family.

Clinical findings

Physical examination revealed tenderness upon palpation of the lower abdomen and bilateral adnexal masses on bimanual pelvic examination. Blood tests were conducted to assess female hormonal levels, such as anti-Müllerian hormone at 0.053 ng/ml, which was lower than the reference limits; follicle-stimulating hormone (FSH) at 3.60 mIU/ml; and luteinizing hormone (LH) at 6.70 mIU/ml. Both LH and FSH were found to be in the normal range, and her cancer antigen 125 levels were 19.00 U/ml, which were within normal limits, as shown in Table 1.

**Table 1:** Results of the female hormonal profile AMH, anti-Müllerian hormone; CA 125, cancer antigen 125; FSH, follicle-stimulating hormone; LH, luteinizing hormone

Hormonal profile	Findings	Reference limits
AMH	0.053 ng/ml	1.5-4.0 ng/ml
LH	6.70 mIU/ml	2-15 mIU/ml
FSH	3.60 mIU/ml	3.5-12.5 mIU/ml
CA 125	19.00 U/ml	≤35.00 U/ml

**Figure 1:**
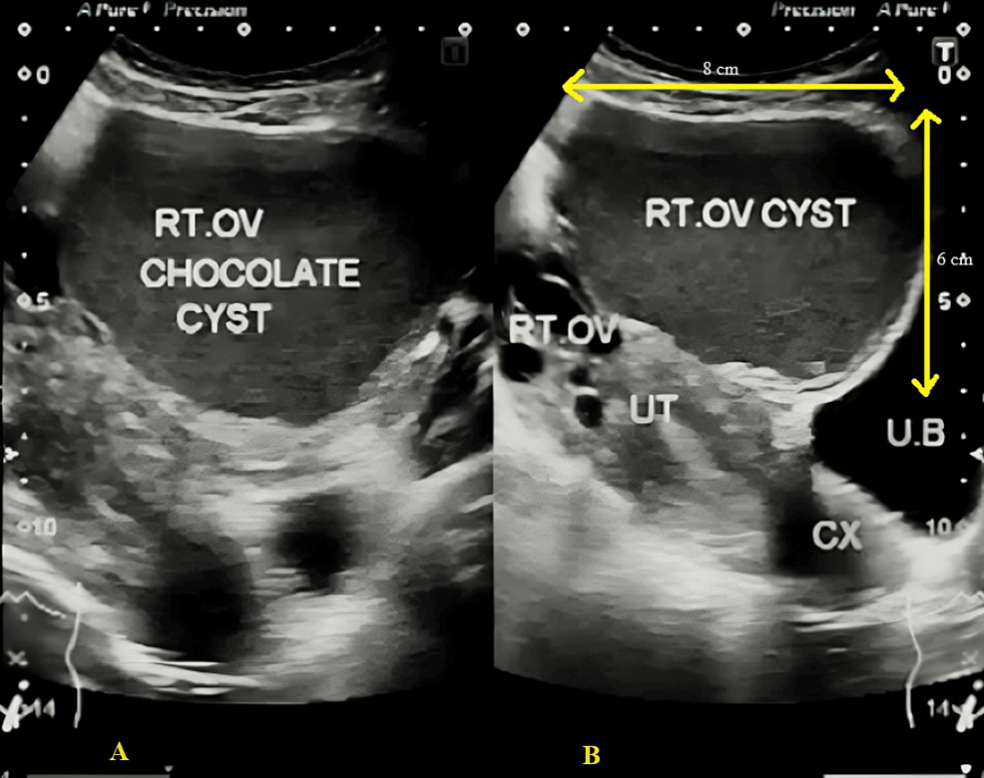
TAUS of the female patient showing chocolate cyst in right ovary (A) Axial image. (B) Sagittal image. CX, cervix; OV, ovary; RT, right; TAUS, transabdominal ultrasound; UB, urinary bladder; UT, uterus

A transvaginal ultrasound was done on day 2 of menses to determine the quantity and quality of the patient’s remaining oocytes. The antral follicle count was within normal limits. The endometrial lining appeared to be thin. A hysterosalpingography procedure was done to assess the health of the female patient’s fallopian tube and the uterine cavity, ensuring there were no structural abnormalities. Transabdominal ultrasound and laparoscopic ultrasound scanning confirmed the presence of endometriosis with adhesions and a chocolate cyst measuring 8 cm × 6 cm in dimensions in the right ovary. Her Ovarian-Adnexal Reporting and Data System (O-RADS) ultrasound stage was less than 1, giving certainty of a benign cyst. The transabdominal ultrasound image of pelvic organs, along with axial and sagittal images, reveals well-defined cystic lesions with dense internal echoes within the right ovary, as shown in Figure 1.

Seminal parameters of her husband were taken. His sperm count was measured at 26 M/ml, with a total motility of 67%, progressive motility of 34%, and a morphological abnormality percentage of 92%. The pH level was recorded at 7.2, which is within the normal range. The volume of the ejaculate was measured at 2.5 ml, as shown in Table 2. All the seminal parameters were based on WHO guidelines [12].

**Table 2:** Semen microexamination report of the patient

Semen parameters	Findings
Abstinence period	Three days
Sperm count	26 M/ml
Total sperm motility	67%
Progressive motility	34%
pH	7.2
Volume	2.5 ml
Morphologically abnormal sperm	92%
Normal morphological sperm	8%

Diagnosis

The patient was provisionally diagnosed with a chocolate cyst, also known as an ovarian endometrioma, which may lead to chronic pelvic pain, dysmenorrhea, and potential fertility challenges due to its impact on ovarian function and structure.

Therapeutic intervention

**Figure 2:**
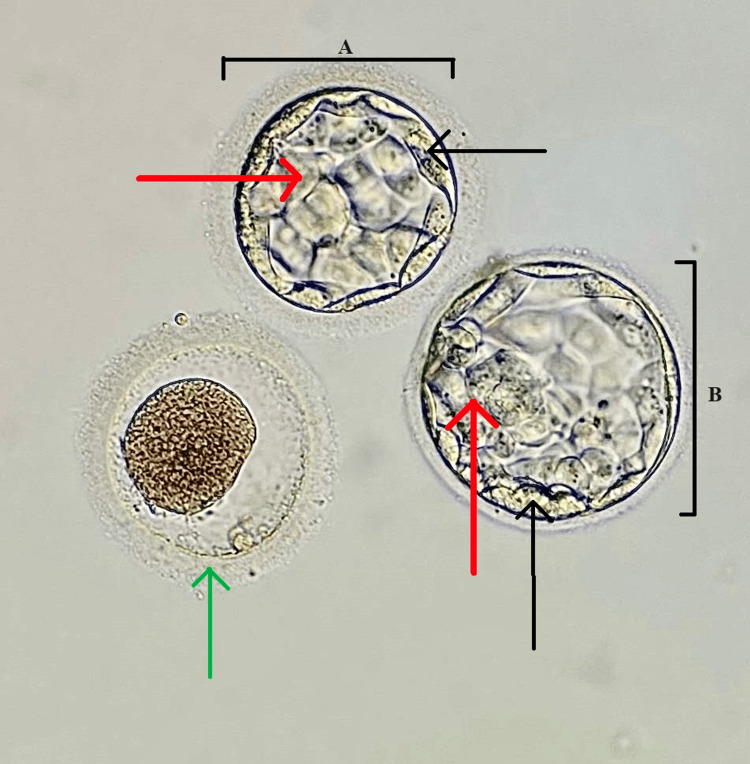
Day 5 blastocysts of grades 3AA and 4AA were transferred during a fresh embryo transfer procedure (A) First blastocyst. (B) Second blastocyst. 3AA: blastocoel fills the blastocyst with numerous and tightly packed cells in ICM and many cells organized in the epithelium of TE; 4AA: expanded blastocyst with numerous and tightly packed cells in ICM and many cells organized in the epithelium of TE Black arrow: TE; red arrow: ICM; green arrow: degenerate oocyte ICM, inner cell mass; TE, trophectoderm

After proper counseling, the patient underwent a laparoscopic cystectomy for ovarian endometriomas. Intraoperatively, chocolate-colored fluid consistent with endometriotic cyst contents was aspirated, and an excision of the cyst walls was performed. Histopathological examination confirmed the presence of endometriotic glands and stroma within the ovarian tissue. Postoperatively, the patient had an uneventful recovery and reported significant improvement in pelvic pain. The patient was prescribed hormonal therapy, which is gonadotropin-releasing hormone (GnRH) antagonists, to stimulate ovarian function and prevent her condition of ovarian hyperstimulation syndrome. Considering the severity of endometriosis, it was recommended that the couple proceed with the second IVF cycle. The female partner underwent an ovarian stimulation protocol. We administered short-acting GnRH antagonists with regular monitoring. We used letrozole at 5 mg per day, and human menopausal gonadotropin at 150 IU was administered in addition to the GnRH agonist trigger. Triptorelin (Decapeptyl) 0.2 mg trigger was given 36 hours before ovum retrieval. On day 13, ovum pickup was done, and only three oocytes from the left ovary were retrieved; all the oocytes were in the metaphase 2 stage. ICSI was done on the same day. After 16 hours of incubation, a fertilization check was done; only two oocytes were fertilized, and two blastocysts of grade 3AA and 4AA were formed, as shown in Figure 2.

On day 17, fresh embryo transfer of grade 2 embryos of the cleavage stage was done. PRP was done on day 14 of menses for better implantation. Endometrial thickness was noted at 9 mm pre-PRP and 12 mm post-PRP on the day of embryo transfer. Two weeks post-embryonic transfer, beta-human chorionic gonadotropin (β-hCG) was 509 mIU/ml, indicating a positive pregnancy.

Follow-up

After 14 days of successful embryo transfer, the urinary pregnancy test was positive, and the serum β-hCG level was 509 mIU/ml. Regular follow-up assessments through ultrasound and hormonal monitoring were performed to evaluate the response to treatment. The patient was instructed to avoid strenuous activity and heavy lifting and to get plenty of rest. The patient also received calciferol sachets for calcium intake, prednisolone (5 mg) calcium, multivitamins, and iron supplements. The patient was advised to keep taking her medications.

## Discussion

Laparoscopic surgery was initially considered to be the best option for the treatment of endometriosis-related infertility [13]. In this case, the significant contributor to infertility is the female patient’s condition of an ovarian cyst, which was removed by laparoscopic excision and advised for an ICSI cycle where she is stimulated by a short gonadotropin antagonist protocol. For better implantation, we administered PRP to improve her endometrial receptivity and increase the chance of implantation, which results in a successful pregnancy. The combination of surgical intervention followed by ART has been demonstrated to provide improved prospects of conception for women experiencing infertility as a result of endometriosis. Nevertheless, it has been emphasized that pelvic surgery for endometriosis, particularly in instances involving ovarian endometriomas, may potentially result in a condition of compromised ovarian reserve, the formation of adhesions, and ischemic damage [14].

Patients undergoing IVF for endometriosis-related infertility respond with significantly lower levels of all reproductive process markers, which almost decrease their pregnancy rate to half compared to women with other IVF indications [15]. PRP intrauterine infusion has been shown to modify immune function at the maternal-fetal interface, increase endometrial receptivity, and improve the ability of embryo implantation in patients with RIF [16]. The treatment approach to infertility and endometriosis has certainly evolved because of the current technological advancements in ultrasound diagnosis as well as in clinical and laboratory aspects of ART [17].

Medical treatment is successful in managing pain and preventing the reappearance of symptoms after surgical removal. However, it is unable to treat infertility. Regardless of the disease’s stage, surgery increases the likelihood of natural conception within the subsequent 12 to 18 months [18]. When considering ART, surgery is ineffective because it does not improve the outcome and can harm the ovarian response to stimulation. Today, ART is frequently the primary option to be considered in women whose infertility is associated with endometriosis, whose ovarian reserve is compromised, and who are over 35 years of age [19].

## Conclusions

The case highlights the complex relationship between endometriosis and infertility, emphasizing the need for a multidisciplinary approach. Laparoscopic surgery is crucial for addressing endometriosis-related infertility, especially in cases involving ovarian cysts. The successful outcome achieved through ART, specifically with ICSI, underscores the importance of technological advancements in overcoming infertility barriers. The ovarian stimulation protocol coupled with PRP perfusion to enhance endometrial receptivity exemplifies the personalized and innovative approaches available to couples undergoing fertility treatment.
